# Simultaneous Irradiation of Fibroblasts and Carcinoma Cells Repress the Secretion of Soluble Factors Able to Stimulate Carcinoma Cell Migration

**DOI:** 10.1371/journal.pone.0115447

**Published:** 2015-01-30

**Authors:** Adnan Arshad, Eric Deutsch, Marie-Catherine Vozenin

**Affiliations:** 1 INSERM U1030, LabEx LERMIT, Villejuif, France; 2 Université Paris Sud, Kremlin-Bicêtre, France; 3 Institut Gustave Roussy, Villejuif, France; 4 Laboratoire de recherche en Radio-Oncologie, Service de Radio-Oncologie/département d’Oncologie/CHUV, Lausanne, Switzerland; Ghent University, BELGIUM

## Abstract

Stroma mediated wound healing signals may share similarities with the ones produced by tumor’s microenvironment and their modulation may impact tumor response to the various anti-cancer treatments including radiation therapy. Therefore we conducted this study, to assess the crosstalk between stromal and carcinoma cells in response to radiotherapy by genetic modulation of the stroma and irradiation. We found that fibroblasts irrespective of their RhoB status do not modulate intrinsic radiosensitivity of TC-1 but produce diffusible factors able to modify tumor cell fate. Then we found that Wt and RhoB deficient fibroblasts stimulated TC-1 migration through distinct mechanisms which are TGF-β1 and MMP-mediated respectively. Lastly, we found that simultaneous irradiation of fibroblasts and TC-1 abrogated the pro-migratory phenotype by repression of TGF-β and MMP secretion. This last result is highly relevant to the clinical situation and suggests that conversely to, the current view; irradiated stroma would not enhance carcinoma migration and could be manipulated to promote anti-tumor immune response.

## Introduction

Wound healing and carcinogenesis are defined as complex, adaptive processes which are controlled by intricate communications between the host and the tissue microenvironment. During a normal wound healing process, regeneration and repair of a wound, depends on a variety of signals which coordinate the response to injury. These processes entail cell proliferation, survival, and migration which are controlled by growth factors, cytokines as well as inflammatory and angiogenic signals. These signals are derived from multiple intra and extracellular components embedded in the microenvironment of wounds and are also involved in cancer. Therefore, a number of phenotypic similarities are shared by wounds and cancers in cellular signaling and gene expression. These similarities between wound healing and carcinogenesis were first recognized by Haddow, and the notion that ‘cancer are wounds that do not heal’ was defined by Dvorak [1, 2].

Radiotherapy is the second most effective modality of cancer treatment after surgery and can be used, either alone or in combination with chemotherapy. The main anti-tumor effect of radiation therapy is the induction of tumor cell death but recent findings suggest that radiotherapy also rapidly and persistently modifies the tissue microenvironment. These modifications affect cell phenotype, tissue metabolism, bidirectional exchanges and signaling events between cells [3]. While there is evidence indicating that these changes might contribute to the antitumor effects of radiotherapy, some clinical and experimental observations indicates that irradiated stroma might exert tumor-promoting effects [3]. MH. Barcellos-Hoff’s Group has indeed shown a major contribution of TGF-β1 produced by irradiated stroma to carcinogenesis [4–6] and high dose of radiotherapy are known to stimulate TGF-β1 production [7]. TGF-β1 is the prototype of pro-wounding molecules shown to be the main inducer of reactive stroma, by not only affecting chemotaxis of fibroblasts, but also their trans-differentiation into reactive fibroblasts, termed myofibroblasts [8]. TGF-β1 also regulates epithelial phenotype and has been especially described as a potent stimulatory molecule during the late phase of carcinogenesis and metastasis dissemination.

Beside TGF-β1 signal, the contribution of the Rho pathway to radiation response has been proposed by our group and others [9]. Rho GTPases are a family of signaling mediators implicated in regulating cytoskeletal dynamics, motility, cell division, and transcriptional regulation. More specifically, RhoB expression is increased by a variety of extra-cellular stimuli which include irradiation, epidermal growth factor (EGF) and transforming growth factor β (TGF- β) [7, 10]. Most Rho proteins are modified by the covalent attachment of a geranylgeranyl group, but RhoB can exist in either a geranylgeranylated (RhoB-GG) or a farnesylated (RhoB-F) form. RhoB-F localizes to the cell membrane, modulates actin cytoskeleton, activates nuclear factor kappa B and promotes cell growth [11–13]. In contrast, RhoB-GG localizes to endosomes and induces cell apoptosis [11]. A role for RhoB in TGF-β induced cell responses (such as epithelial-mesenchymal transition (EMT) and apoptosis ) was suggested by a series of DNA microarray studies, which showed that RhoB expression was upregulated by TGF-β in a variety of cell types such as keratinocytes, mouse mammary gland epithelial cells, hepatoma cells, and dermal fibroblasts [14]. TGF-β also stimulates actin stress fiber formation in Ras-transformed cells in a way which is associated with upregulation of RhoB [15, 16]

In the present study, our hypothesis was that scarring signals including TGF-β and RhoB that are activated by irradiation in the stroma could enhance tumor aggressiveness after radiation therapy. Therefore, RhoB deficiency would indirectly enhance anti-tumor effect of radiation therapy. To investigate this crosstalk, we used *in vitro* a co-culture model consisting of lung carcinoma cells and lung primary fibroblasts. We also used RhoB deficient fibroblasts to assess our hypothesis. Interestingly we found that the intrinsic radiosensitivity of carcinoma cells was not modulated by paracrine factors secreted either by Wt or RhoB-/- fibroblasts. Irradiation of fibroblasts stimulated migration of carcinoma cells but simultaneous irradiation of carcinoma and fibroblasts repressed the secretion of these pro-invasive signals by fibroblasts.

## Materials and Methods

### 1- Cells

C57BL6 (Wt) and RhoB-/- mice [16,17] were used to isolate primary lung fibroblasts by enzymatic digestion (collagenase/trypsin) and cells were subcultured in DMEM +Glutamine with 20% foetal calf serum (FCS), 50U penicillin /streptomycin, 1% Hepes,10mg EGF, ITS. RhoB deficiency was controlled and monitored by genotyping and western-blot. C57BL6 mice were purchased from Charles River laboratories, RhoB mice were obtained from Pr Prendergast GC laboratory. Mice were maintained in the animal care facilities of Institut Gustave-Roussy (agreement No. D94076-11). Authorization for experiments was obtained from the Comité d’Ethique en Expérimentation Animale Paris-11.

TC-1 cells (Murine adenocarcinoma lung) were grown in RPMI 1640 medium with 10% fetal bovine serum or conditioned medium isolated from fibroblasts culture.

### 2- Chemicals

The Inhibitor of TGF-β type I receptor (ALK5, ALK4 and ALK7), SB 431542 was used at 10 μM and the MMP Inhibitor, O-Phenanthroline at 100 μM. Both were bought from Santa Cruz.

### 3- Culture with Conditioned Medium (CM)

**Figure 1:**
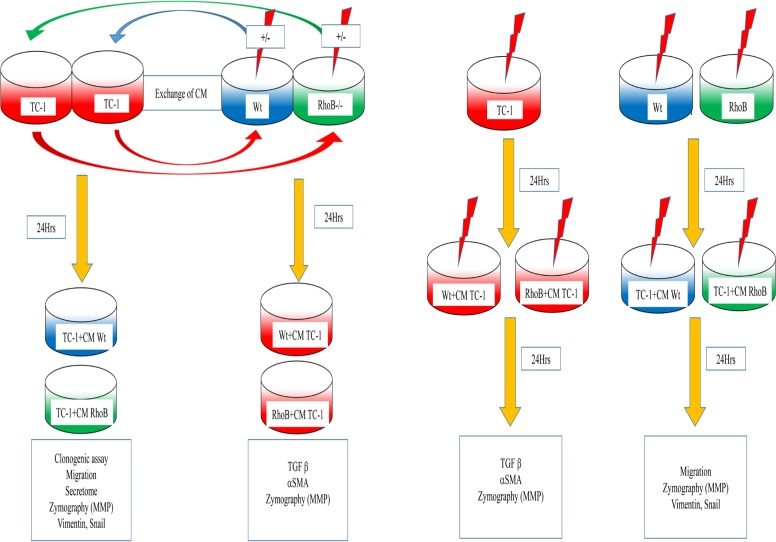
Experimental Scheme. **A) Step 1** Wt and RhoB-/- fibroblasts are cultured at subconfluence and irradiatedTC-1 are also cultured at subconfluency. **Step 2** After 24hrs, Conditioned media (CM) produced by the three type of cells irradiated or not are collected. CM from irradiated or Wt and RhoB -/- fibroblasts are used to culture TC-1 CM from TC-1 is used to culture Wt and RhoB-/- fibroblasts **Step 3** 24hrs later various experiments are performed. **B)** For simultaneous irradiation experiments. ***Left panel* Step 1** TC-1 Cells are irradiated **Step 2** 24h later irradiation TC-1 medium is replaced by the CM produced by irradiated Wt or RhoB-/- fibroblasts **Step 3** 24 h later experiments are performed: ***Right panel* Step 1** Wt and RhoB -/- fibroblasts are irradiated **Step 2** 24h later irradiation fibroblasts medium is replaced by the CM produced by irradiated TC-1 **Step 3** 24 h later experiments are performed.

Briefly, fibroblasts and TC-1 cells were cultured into their preferred medium as described. Then 24Hrs before experiments medium was changed to 50% RPMI + 50% DMEM with penicillin /streptomycin, ITS and Hepes. This medium supports normal growth of both TC-1 cells and fibroblasts. The same medium is used for all conditions (non-irradiated/irradiated). The various experimental conditions are summarized in Fig. 1A and 1B. After culture with conditioned medium (CM) supernatants and cells were prepared for analysis as indicated.

### 4- Irradiation

TC-1, Wt and RhoB-/- fibroblasts were grown to 80% confluence, culture medium was replaced with FCS-free medium before irradiation. TC-1 and fibroblasts were irradiated with Cs137 (IBL-637 (CIS-BioInternational, France) gamma irradiator (dose rate 1Gy/Min) at indicated dose.

### 5- Clonogenic Assay

TC-1 cells were plated at different cellular density. 4h after seeding, cells were irradiated at doses ranging from 0 to 10 Gy (Cesium = Cs137, 1 Gy/min, gamma irradiator IBL-637 from CIS-BioInternational, IBA, Saclay, France). Cells were incubated for 5–7 days under standard culture conditions and colonies were stained with crystal violet, washed and counted. The same is done with conditioned medium added from non irradiated/irradiated Wt and RhoB -/- fibroblasts to TC-1 cells irradiated/non irradiated. Colonies with more than 50 cells were counted and surviving fraction (SF) for each was calculated by dividing the number of cell clones by the number of cells plated and was normalized to the ratio of clonogenic survival of non-irradiated controls and clonogenic survival for each radiation dose.

### 6- Wound Healing Assay

Wild type (Wt), RhoB-/- Fibroblasts and TC-1 cells were grown to confluence in 6-well culture plates. Cells were irradiated at 10Gy. 24 hours later cell layers were wounded using a sterile 200 μl pipette tip and dead cells are washed out, then conditioned medium was added. Images are captured at 0hr with Nikon Phase contrast, Japan at 10× and plates were returned to the incubator to recover from wounding. After 24 Hrs culture plates were removed and monolayers were photographed again. Wound width was measured on hard copy prints of the images.

### 7- Electrophoresis and Western-Blotting

Cell lysate are prepared with RIPA lysis buffer (Sigma Biotech). Protein concentration was measured using a BioRad protein assay and proteins were submitted to electrophoresis. Primary antibodies were diluted in TBS-T solution (Vimentin (E-5) dil 1:1000 from Santa Cruz Biotechnology, Snail (C15D3) 1:1000, TGF β1(V) 1:250 all from Cell Signaling, αSMA 1:500 from Abcam and GAPDH 1:5000 from Millipore overnight at 4°C, washed next day in TBS-T and incubated with the horseradish peroxidase-conjugated anti-mouse or anti-rabbit secondary antibody. The blots are developed using SuperSignal West Pico Chemiluminiscent Substrate (Pierce Biotechnology, IL) according to the manufacturer’s instructions. The chemiluminiscence signal from the membranes was detected and evaluated using G-box iChemi XT4 digital imaging device (Syngene Europe, Cambridge). Quantification was performed using Image J software. Relative proteins expression are normalized to the respective value for GAPDH.

### 8- Cytokine Array Analysis

Conditioned medium (undiluted) was probed for secreted cytokine profiling using the RayBio Mouse Cytokine Antibody Array 3 and 4 kits according to the manufacturer’s instructions (RayBiotech; Norcross, GA, USA). Quantification was done by Genetools from Syngene. Data were imported into an Excel spreadsheet, normalized against a control across membranes and final values were calculated

### 9- Zymography

Zymography was performed on CM using Novex Zymogram Gels (Invitrogen) following the manufacturer’s protocol as described previously [18].

### 10- Statistical Analysis

Data obtained is the result of 3 independent experiments done in duplicate. Statistical analysis was performed using Graphpad prism 5. Statistical analysis were expressed as mean ± SEM and analyzed using the ANOVA and the Student Newman Keul’s test with a p value < 0.05 was considered significant.

## Results

### 1. Paracrine Signals Secreted By Fibroblasts do not modulate TC-1 Radiosensitivity

**Figure 2:**
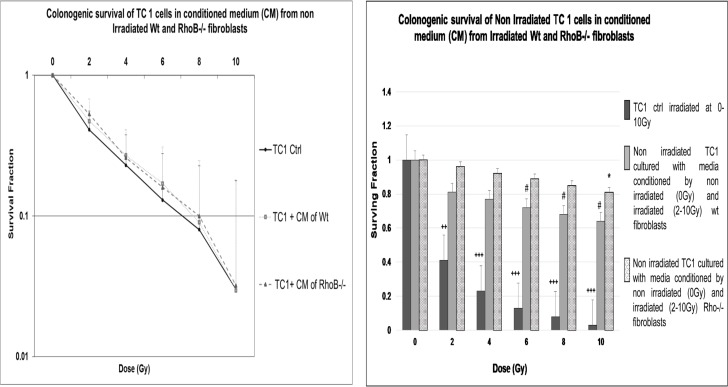
TC-1 Clonogenic Survival Curve: Paracrine signals secreted by fibroblasts do not modulate TC-1 intrinsic radiation sensitivity. **A)** Media conditioned by fibroblasts do not modify TC-1 intrinsic radiation sensitivity. **B)** Conditioned Media (CM) isolated from irradiated Wt and RhoB-/- fibroblasts increase the clonogenic potential of non-irradiated TC-1 cells.

To test whether paracrine signals secreted by fibroblasts would have an effect on the intrinsic radiosensitivity of TC-1 cells, we produced conditioned medium from Wt and RhoB -/- fibroblasts and used them to culture TC-1 in clonogenic survival assay. Fig. 2A showed that media conditioned by fibroblasts (irrespective of their genotype) do not modify TC-1 intrinsic radiation sensitivity assessed by clonogenic assays, suggesting that signals triggered by irradiation are stronger than survival signals triggered by fibroblasts.

Interestingly, CM isolated from irradiated Wt and RhoB-/- fibroblasts do alter clonogenicity and survival of non-irradiated TC-1, suggesting that paracrine signals are produced by irradiated fibroblasts and alter TC-1 clonogenic potential (Fig. 2B).

### 2. Paracrine Signals secreted by fibroblasts enhance TC-1 migration

**Figure 3:**
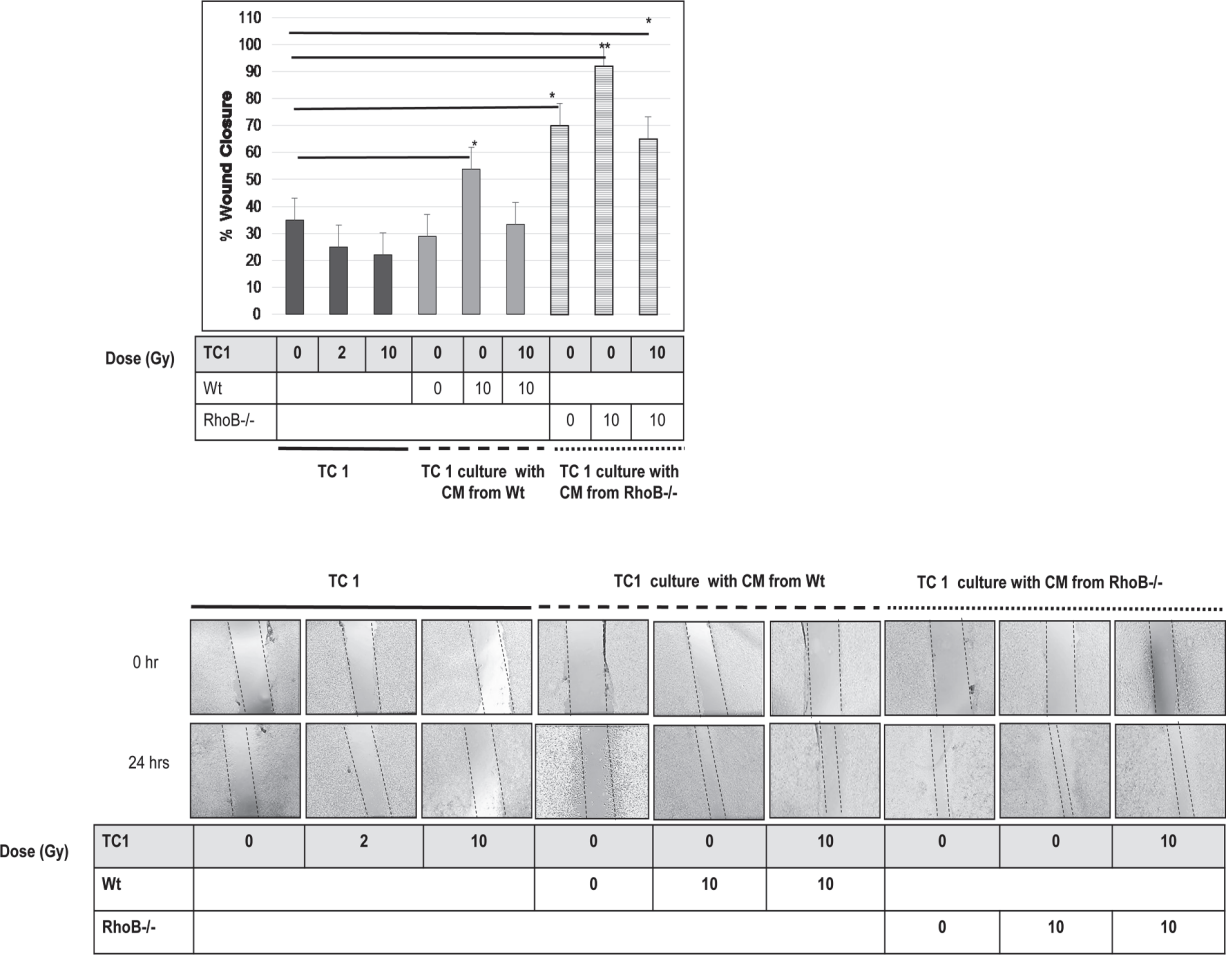
Paracrine Signals secreted by fibroblasts enhance TC-1 migration. Migratory potential of TC -1 cells was analyzed and quantified in various culture conditions at 0 and 24 Hrs after wounding. Culture conditions are: cultured TC-1 non-Irradiated (0Gy) and Irradiated at 2, 10 Gy alone or cultured using conditioned media from Wt and RhoB -/- fibroblasts. Images are recorded at x20 with Nikon Phase contrast, Japan. In all cases, differences were considered significant at: * P<0.05; ** P<0.01; *** P<0.001.

We assessed whether irradiation modulated TC-1 cell motility and showed that TC-1 migration reduced with increasing dose of irradiation. Then, we showed that conditioned medium (CM) produced by non-irradiated Wt fibroblasts had no effect on TC-1 cell migration, whereas CM produced by 10 Gy irradiated fibroblasts significantly stimulated TC-1 cells migratory capability (Fig. 3A). Interestingly when TC-1 are irradiated and cultured in CM produced by 10Gy irradiated Wt fibroblasts (Fig. 3B), TC-1 cell migration returned to normal level. Alongside we showed that CM produced by non-irradiated RhoB-/- fibroblasts increased TC-1 cell migration more significantly than Wt fibroblasts, suggesting that RhoB deficiency promote the production of pro-migratory signals. This stimulation is further enhanced by 10 Gy irradiated RhoB-/- fibroblasts (Fig. 1A) but repressed back to the control level when TC-1 are irradiated at 10 Gy and cultured in CM produced by irradiated RhoB -/- fibroblasts (Fig. 1B, 3A and 3B).

### 3. TC-1 migration is mediated by MMP

**Figure 4:**
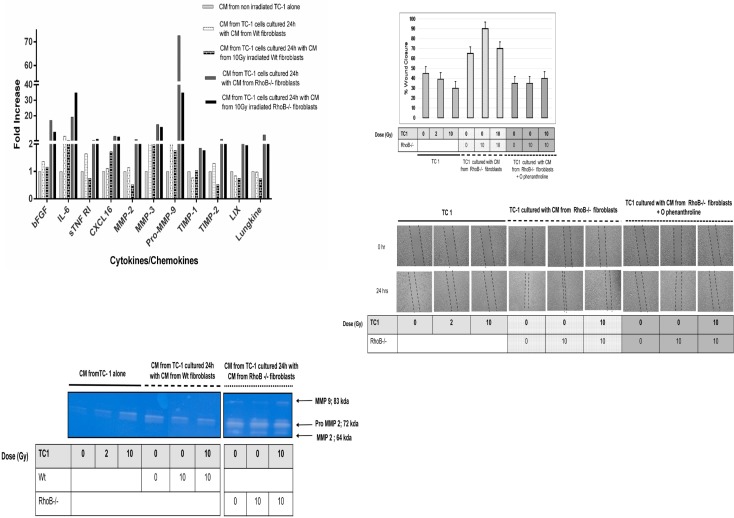
**A) Cytokine Array Analysis:** performed on TC-1 secretome to search for mediators of Increase TC-1 invasiveness. Significantly upregulated cytokines are shown. **B) MMP secretion from TC 1 cells**. Zymography was performed on conditioned media collected from TC-1 cells 24h after culture with conditioned media from Wt and RhoB-/- fibroblasts. **C) Modulation of TC-1 migration by O-Phenanthroline**. Migratory potential of TC-1 cells was analyzed and quantified in various culture conditions at 0 and 24 h after wounding. Culture conditions are: CM from TC-1 non Irradiated (0Gy) and irradiated at 2, 10 Gy alone or cultured with CM from Wt fibroblasts with O-Phenanthroline. Images are acquired at x20 with Nikon Phase contrast, Japan. In all cases, differences were considered significant at: * P<0.05; ** P<0.01; *** P<0.001.

To search for mediators of TC-1 cell invasiveness and migration, we investigated the secretome of TC-1 cultured alone or cultured for 24h with CM produced by Wt and RhoB-/- fibroblasts irradiated or not. While most tested proteins (96) were not affected, production of IL-6, bFGF, CXCL 16, sTNF RI, MMP 2, MMP 3, Pro MMP 9 were significantly stimulated when TC-1 were cultured with fibroblast’s CM (Fig. 4A). Interestingly, CM from RhoB deficient fibroblasts modulated more proteins secreted by TC-1 than CM from Wt, with a marked increase in Pro MMP-9, MMP-3 and MMP-2.

We confirmed MMP induction by zymogram analysis. Our results show a weak secretion of MMP 2 and MMP 9 when TC-1 are cultured alone, slightly enhanced when TC-1 are irradiated (10 Gy). CM of Wt fibroblasts irradiated or not does not change the level and activity of MMP2/9 produced by TC-1, whereas CM from RhoB-/- fibroblasts irradiated or not significantly enhance level and activity of proMMP2, MMP2 and MMP9 (Fig. 4B) in TC-1 secretome. This suggests that RhoB deficient fibroblasts secrete MMPs and/or paracrine factors, that remain to be identified, but that are able to enhance MMPs secretion in TC-1. As MMPs are known to degrade the extracellular matrix, we postulated that they could be involved in the enhanced migration of TC-1 and confirmed this hypothesis using the MMP inhibitor O-phenanthroline. Our results show that O-phenanthroline inhibited TC-1 migration cultured with CM medium produced by RhoB -/- fibroblasts irradiated or not (Fig. 4C). Note that in RhoB deficient fibroblasts, the secretion of MMP2 and 9 was not modulated by irradiation nor by CM produced by TC-1 (S1 Fig.), supporting the fact that MMP induction was TC-1 mediated.

### 4. TGF-β1 is secreted by Wt but not RhoB -/- fibroblasts

**Figure 5:**
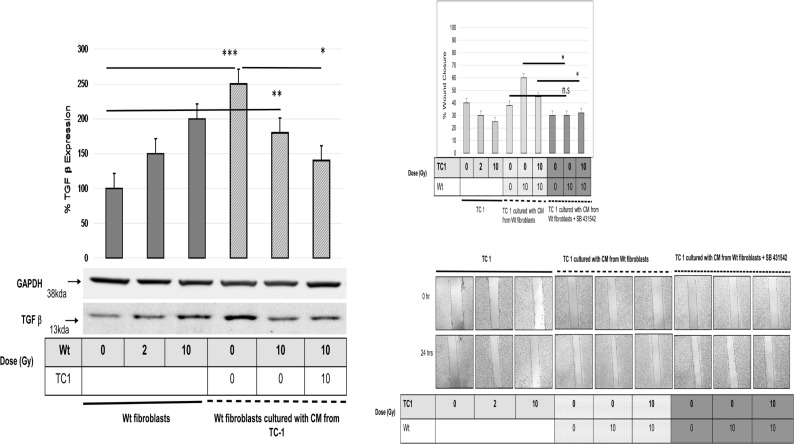
A) Effect of Irradiation and culture with CM on TGF-β Expression in Wt fibroblasts. Whole cell lysate from Wt Fibroblasts was subjected to Western Blot using antibodies for TGF-β1 (13 kDa). Histogram shows relative protein levels normalized to the intensity of the corresponding GAPDH values. In all cases, differences were considered significant at: * P<0.05; ** P<0.01; *** P<0.001. **B) Modulation of TC-1 migration by SB 41542**. Migratory potential of TC-1 cells was analyzed and quantified in various culture conditions at 0 and 24 Hrs after wounding. Culture conditions are: CM from TC-1 non Irradiated (0Gy) and irradiated at 2, 10 Gy alone or cultured with CM from Wt fibroblasts with SB41542. Images are acquired at ×20 with Nikon Phase contrast, Japan. In all cases, differences were considered significant sat: * P<0.05; ** P<0.01; *** P<0.001.

Then we aimed to identify the pro-invasive mediators produced by Wt and RhoB-/- fibroblasts upon irradiation and found that TGF-β1 production was indeed stimulated by irradiation in a dose dependent manner in Wt fibroblast but not in RhoB-/- fibroblasts (S2 Fig.). When Wt Fibroblasts were cultured with CM collected from TC-1, TGF-β1 production by Wt fibroblasts was further enhanced, whereas when Wt fibroblasts were irradiated and cultured with CM produced by 10Gy irradiated TC-1 TGF-β production was repressed (Fig. 1B and 5A). We further confirmed the role of TGF-β1 in accelerated wound closure of TC-1 cells by utilizing SB-431542, a TGF-β inhibitor. We noticed that SB-431542 inhibited Wt fibroblasts induced TC-1 cell motility and delayed the wound closure (Fig 5B) whereas, RhoB-/- induced TC-1 cell motility remained unaffected (S3 Fig.)

### 5. TGF-β1 production induces EMT markers in TC-1

**Figure 6:**
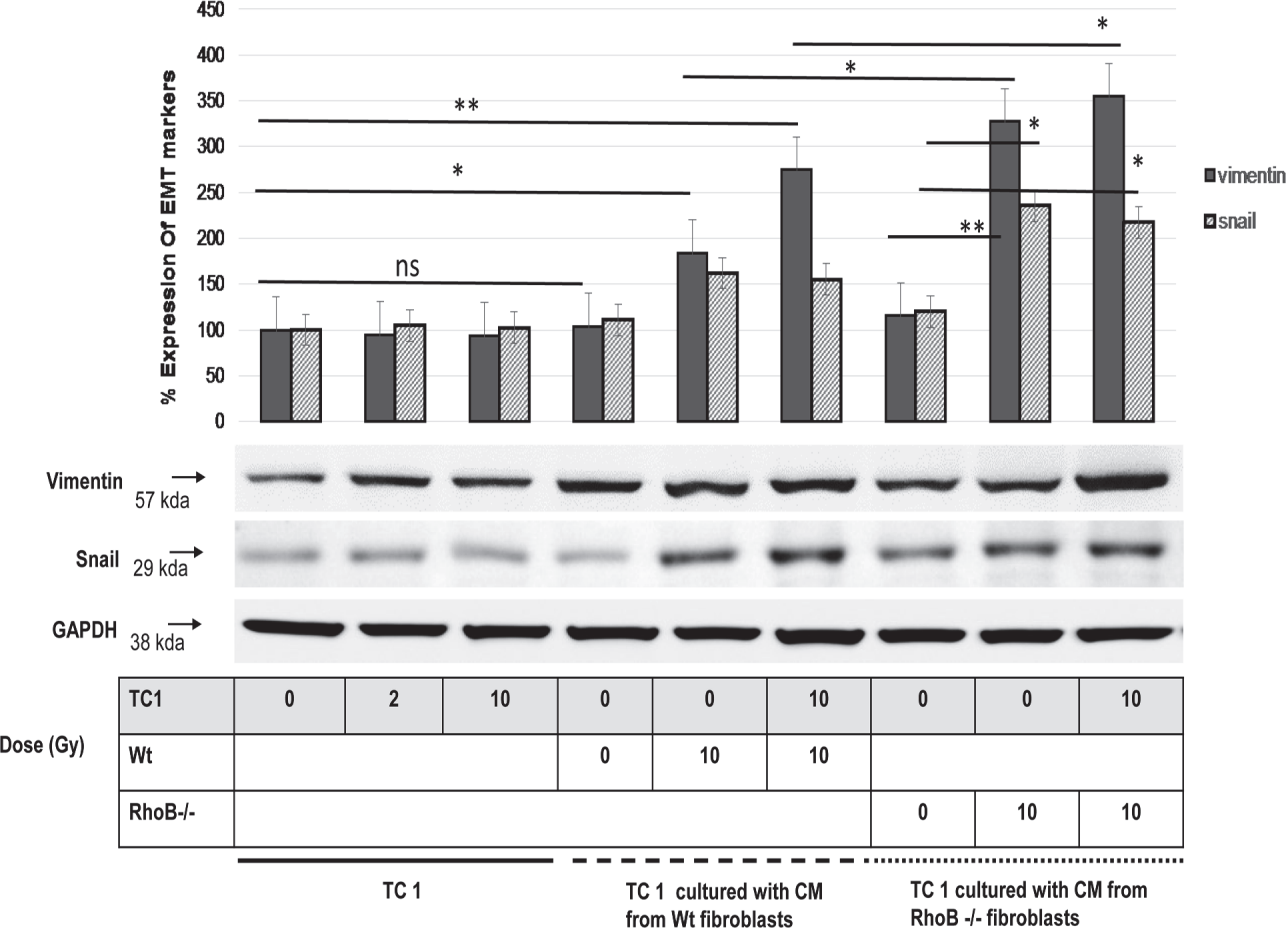
TC-1 enhanced migration is associated with induction of EMT markers: TC-1 lysate was subjected to Western Blot using antibodies for Vimentin (57kDa) and Snail (29kDa). Histograms show the relative protein levels of each normalized to the intensity of the corresponding GAPDH values. In all cases, differences were considered significant at: * P<0.05; ** P<0.01; *** P<0.001.

Then, we investigated whether the variation of migratory potential of TC-1 cells was associated with altered phenotype and induction of two EMT makers, Vimentin and Snail, in carcinoma cells. Fig. 6 showed that ionizing radiation does not promote Vimentin and Snail expression in TC-1 nor does the CM from Wt fibroblasts supporting the Scratch assay’s results (Fig. 3). However, CM produced by 10 Gy irradiated fibroblasts stimulated both Vimentin and Snail protein expression in TC-1 suggesting induction of an EMT phenotype by paracrine factors secreted by irradiated fibroblasts. Surprisingly, simultaneous irradiation of Wt fibroblast and TC-1 further enhanced Vimentin expression but has no effect on Snail, suggesting that the decreased migration observed by scratch assay was not associated with alteration of the EMT phenotype.

CM from RhoB-/- fibroblasts do not induce Vimentin nor Snail protein expression in TC-1 suggesting that the enhanced migration observed by scratch assay is mediated by another mechanisms. However when irradiated, RhoB-/- fibroblasts stimulate Vimentin and Snail in TC-1. As for Wt fibroblast, simultaneous irradiation of RhoB-/- fibroblast and TC-1 further enhance Vimentin expression but has no effect on Snail (Fig. 6). These results suggest that various and independent paracrine factor are produced by irradiation of fibroblasts and independently modulate migration and EMT phenotype.

## Discussion

**Figure 7:**
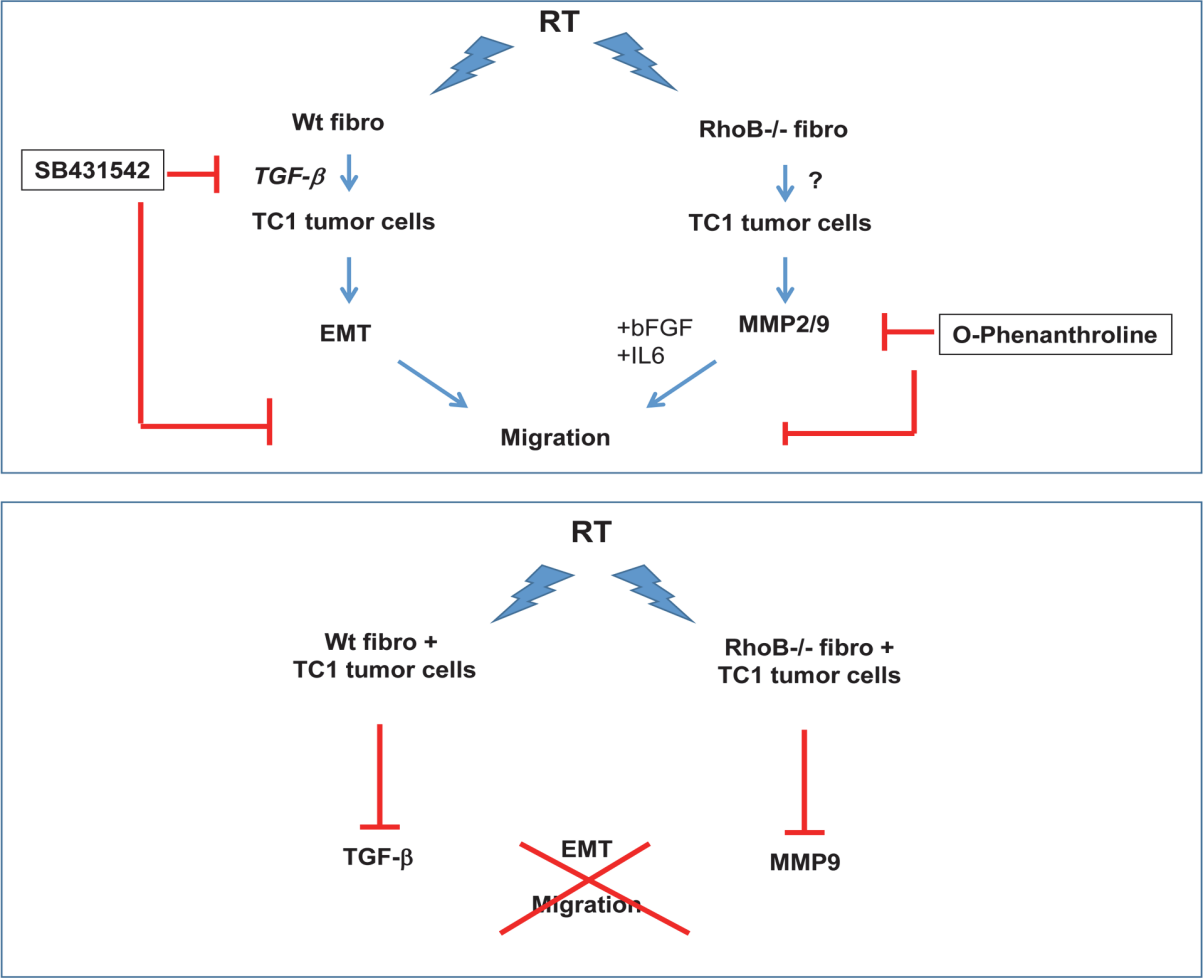
Summary of the findings.

In our present study, we investigated the crosstalk between stromal and carcinoma cells after irradiation and postulated that fibroblasts would promote TC-1 tumor migration after irradiation whereas deficiency in RhoB a protein described to be profibrogenic would prevent it. Our data are different from our initial hypothesis as RhoB deficient fibroblasts enhanced TC-1 migration more potently than Wt fibroblasts do. RhoB deficient fibroblasts stimulated MMP secretion in TC-1 cells thus favoring their migration, whereas Wt fibroblasts stimulated TC-1 migration via TGF-β1 and induction of EMT in TC-1 cells (Fig. 7). We also found that TC-1 intrinsic radiation sensitivity was not altered by conditioned-medium (CM) produced by Wt and RhoB deficient fibroblasts but that clonogenic potential of TC-1 was impaired by diffusible factors secreted by irradiated fibroblasts. Lastly, we found that paracrine factors secreted by irradiated TC-1 tumor cells when cultured in media conditioned by irradiated fibroblasts inhibited both TGF-β1 and MMP induction and repressed their migration.

Our results show that intrinsic radiosensitivity of carcinoma cells TC-1 is not altered by paracrine mediators secreted by non-irradiated stroma but show that the secretion of soluble factors by irradiated stromal cells promotes TC-1 migration and potential metastatic escape. This result is consistent with the literature [19, 20] and as expected we found that TGF-β1 was a stimulatory factors produced by irradiated Wt fibroblast. MH. Barcellos-Hoff’s Group has demonstrated a major contribution for TGF-β1 produced by irradiated stroma to carcinogenesis [21]. TGF-β1 is also known to be the main inducer of reactive stroma and promote chemotaxis of fibroblasts and their transdifferentiation into myofibroblasts [22] by turning on expression of alpha-smooth muscle actin (αSMA). The presence of myofibroblasts-like cells, called CAF, in the tumor stroma may provide a source of growth factors, but could also be involved in connective tissue remodeling allowing expansion and invasion of tumors [23]. In addition, TGF-β1 immunosuppressive function may promote tumor growth and expansion by induction of immune anergy in tumor’s stroma [24].

The use of primary lung fibroblasts isolated from RhoB deficient transgenic mice allowed us to identify activation of 3 major proteins in the secretome of TC-1 tumor cells cultured with conditioned media produced by these fibroblasts: MMP, bFGF and IL-6. Production of MMP3, 9 and bFGF were specifically found to be stimulated in tumor cells when cultured with media conditioned by RhoB -/- fibroblasts but repressed when RhoB -/- fibroblasts were irradiated. As suggested by the literature [25], we postulated that this increased expression of MMPs was associated with tumor invasion and indeed MMP inhibition in co-culture conditions repressed TC-1 migration as assessed in wound closure assay. Beside modulation of the migratory properties of cancer cells, RhoB -/- fibroblasts seemed to modulate both vascular remodeling and immune infiltration signals in tumor cells. Both MMP9 and bFGF are indeed involved in the regulation of vascular function after high doses of irradiation: MMP9 regulates vasculogenesis and recruitment of myeloid cells [26] whereas bFGF is a major pro-angiogenic factor [27, 28]. Our co-culture system does not allow direct assessment of the vascular and immune contribution: Therefore, additional *in vivo* experiments in immuno-competent mice will be needed to conclude. However, the notion that stromal cells could promote an immunostimulating milieu is further supported by our simultaneous finding of IL-6 induction whose secretion is stimulated in TC-1 cells when they are cultured with medium conditioned by RhoB-/- fibroblasts and further enhanced when tumor cells are cultured in conditioned medium by irradiated RhoB-/- fibroblasts. IL-6 is also upregulated by culture with media conditioned by Wt fibroblast but to a lesser extent. IL-6 is one of the best-characterized pro-inflammatory cytokines [29, 30] and our results strongly suggest that after high dose of radiation stromal cells are able to modify the milieu and induce tumor cells to secrete factors able to alter immune infiltrate. Whether we can modify stromal response and peri-tumoral milieu to trigger immuno-stimulatory (IL-6) vs immune-suppressive (TGF-β) response needs to be investigated in detail but our results suggest that RhoB targeting could be a mean to achieve this goal.

Lastly and more interestingly, our results bring a completely new vision and suggest that paracrine factors produced by simultaneous irradiation of tumor cells and fibroblasts abrogate migration of tumor cells. This is more relevant to the clinical situation and in accordance with clinical findings, but challenges previous publication including Ohuchida *et al*. and Hwang *et al.* papers [19, 20] who showed that coculture with irradiated fibroblasts enhanced the invasive potential of pancreatic cancer cells. They concluded that tumor/stroma interactions would stimulate metastasis after irradiation. Although we confirmed that irradiation of fibroblasts (irrespective of their genotype but via distinct mechanisms) promotes TC-1 invasive potential, this set-up is poorly relevant to the clinical situation, as stroma is never irradiated alone but on the contrary simultaneous irradiation of tumors and surrounding stroma is always performed with sufficient safety margins. Therefore and to stick to the clinical situation, we simultaneously irradiated fibroblasts and TC-1 carcinoma cells and in that case our results bring to a different conclusion as we show that simultaneous irradiation of tumor cells and fibroblasts repressed the pro-migratory signals, suggesting that conversely to what was previously described radiotherapy rather prevented metastatic spread than promoted it.

In conclusion, stromal component of the tumor do secrete pro-migratory factors as function of their genotype. Wt fibroblasts pro-migratory action is mainly TGF-β mediated whereas RhoB deficient fibroblasts stimulate MMP secretion by TC-1. In addition and interestingly RhoB deficiency in the stroma enhanced tumor cell migration but simultaneously stimulated pro-inflammatory signals (IL-6) that would impact on immune recruitment and favor anti-tumor immune response. Lastly, our results challenges the view that irradiated stroma would promote migration of carcinoma cells as we show that independently from their genotype paracrine factors secreted after simultaneous irradiation of fibroblasts and carcinoma cells repressed carcinoma cell migration.

## Supporting Information

S1 Fig MMP secretion from RhoB-/- Fibroblasts. Zymography was performed on conditioned media collected from RhoB-/- fibroblasts, 24hrs after culture with CM from TC-1 cells.
(TIF)

S2 Fig Effect of Irradiation and culture with CM on TGF-β Expression in RhoB-/- fibroblasts. Whole cell lysate from RhoB-/- Fibroblasts was subjected to Western Blot using antibodies for TGF-β1 (13 kDa). Histogram shows relative protein levels normalized to the intensity of the corresponding GAPDH values. In all cases, differences were considered significant at: * P<0.05.
(TIF)

S3 Fig Modulation of TC-1 migration by SB 41542. Migratory potential of TC-1 cells was analyzed and quantified in various culture conditions at 0 and 24 Hrs after wounding. Culture conditions are: CM from TC-1 non Irradiated (0Gy) and irradiated at 2, 10 Gy alone or cultured with CM from RhoB -/-fibroblasts with SB41542. Images are acquired at x20 with Nikon Phase contrast, Japan. In all cases, differences were considered significant at: * P<0.05; ** P<0.01; *** P<0.001.
(TIF)
