# Supplementary figures and images for: Simultaneous Irradiation of Fibroblasts and Carcinoma Cells Repress the Secretion of Soluble Factors Able to Stimulate Carcinoma Cell Migration
Source: PLoS One. 2015 Jan 30;10(1):e0115447. doi: 10.1371/journal.pone.0115447 (PMC4312053; doi:10.1371/journal.pone.0115447)

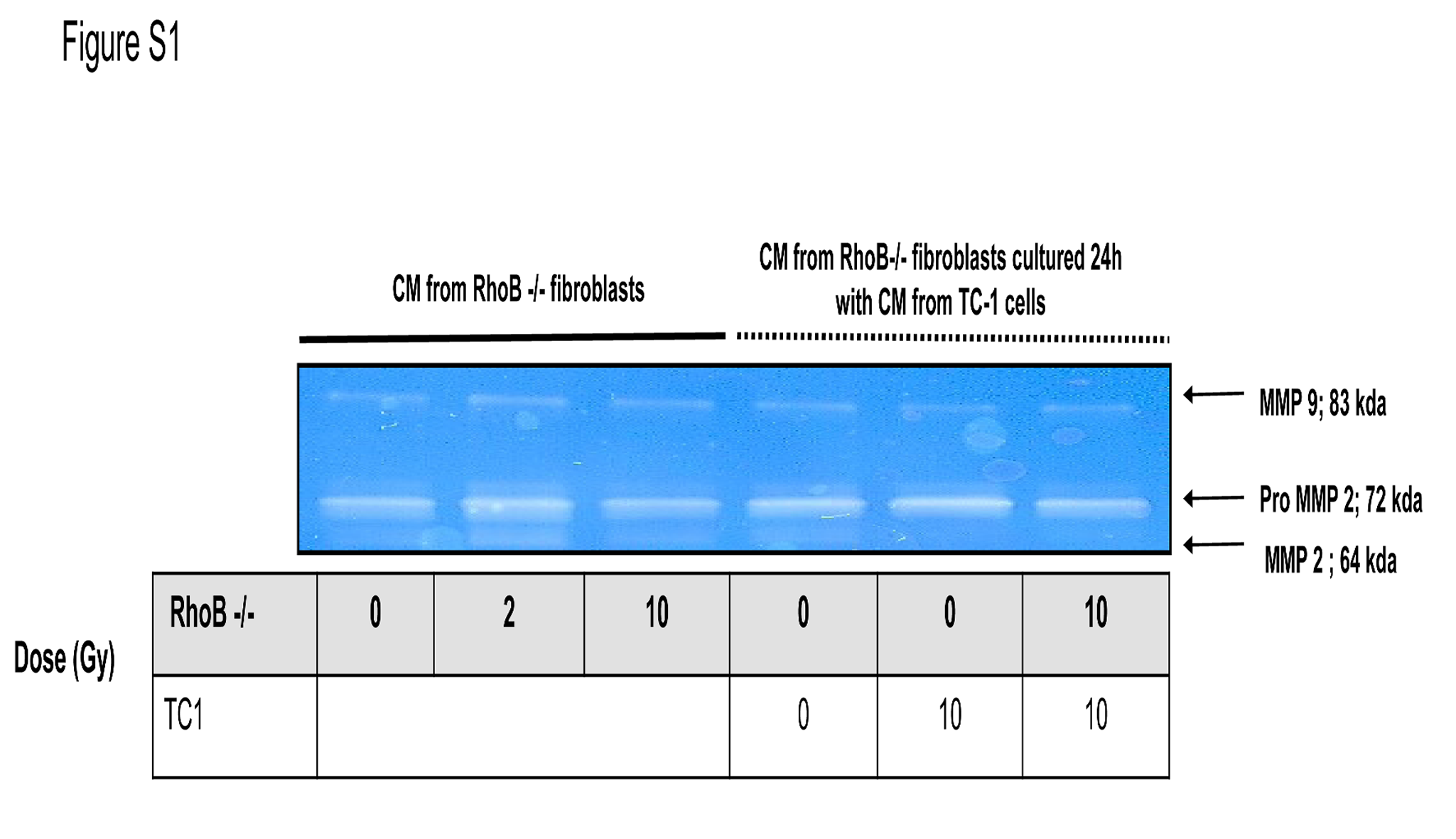

Supplement: S1 Fig — Zymography was performed on conditioned media collected from RhoB-/- fibroblasts, 24hrs after culture with CM from TC-1 cells. (TIF) [file pone.0115447.s001.tif]

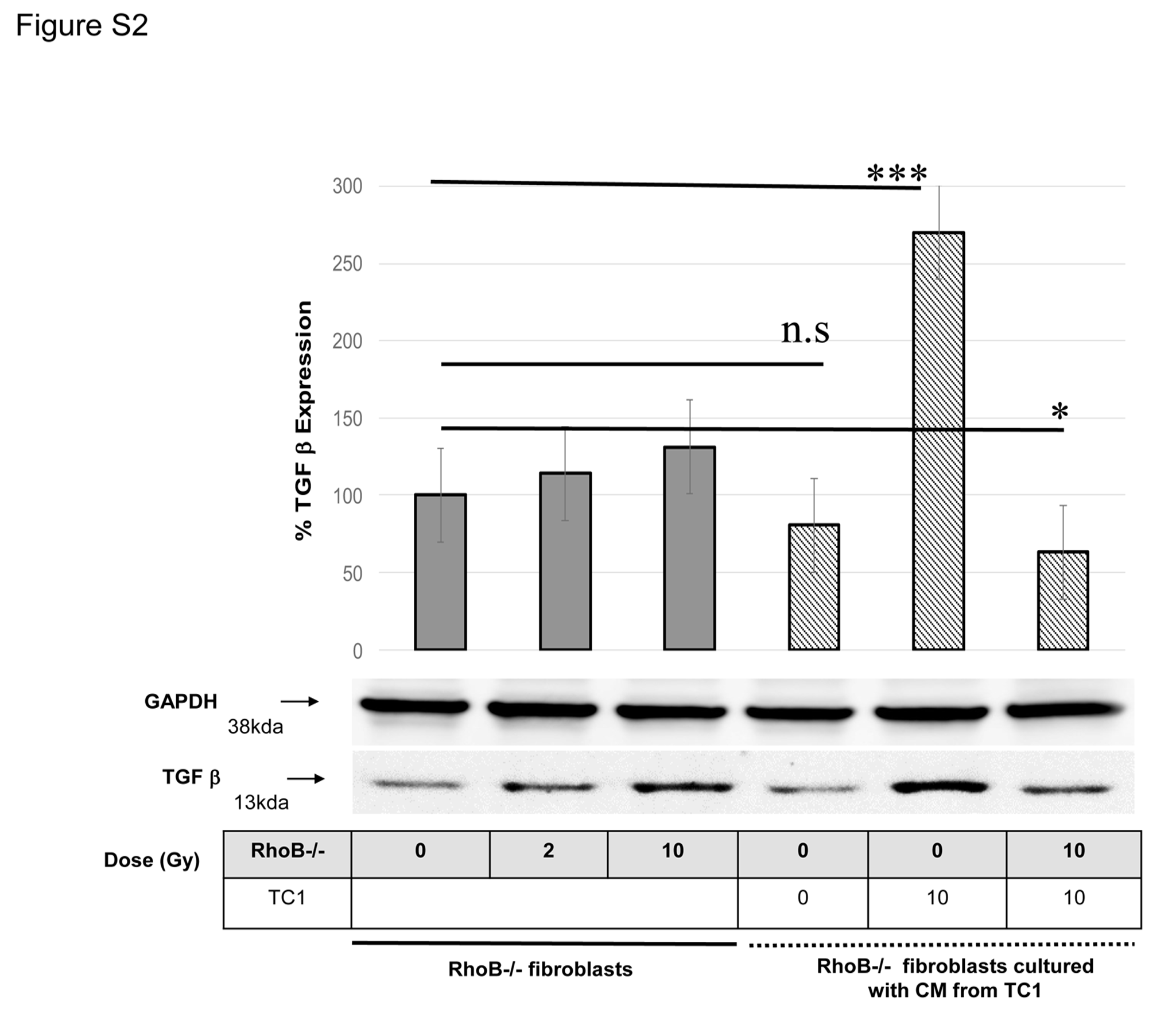

Supplement: S2 Fig — Whole cell lysate from RhoB-/- Fibroblasts was subjected to Western Blot using antibodies for TGF-β1 (13 kDa). Histogram shows relative protein levels normalized to the intensity of the corresponding GAPDH values. In all cases, differences were considered significant at: * P<0.05. (TIF) [file pone.0115447.s002.tif]

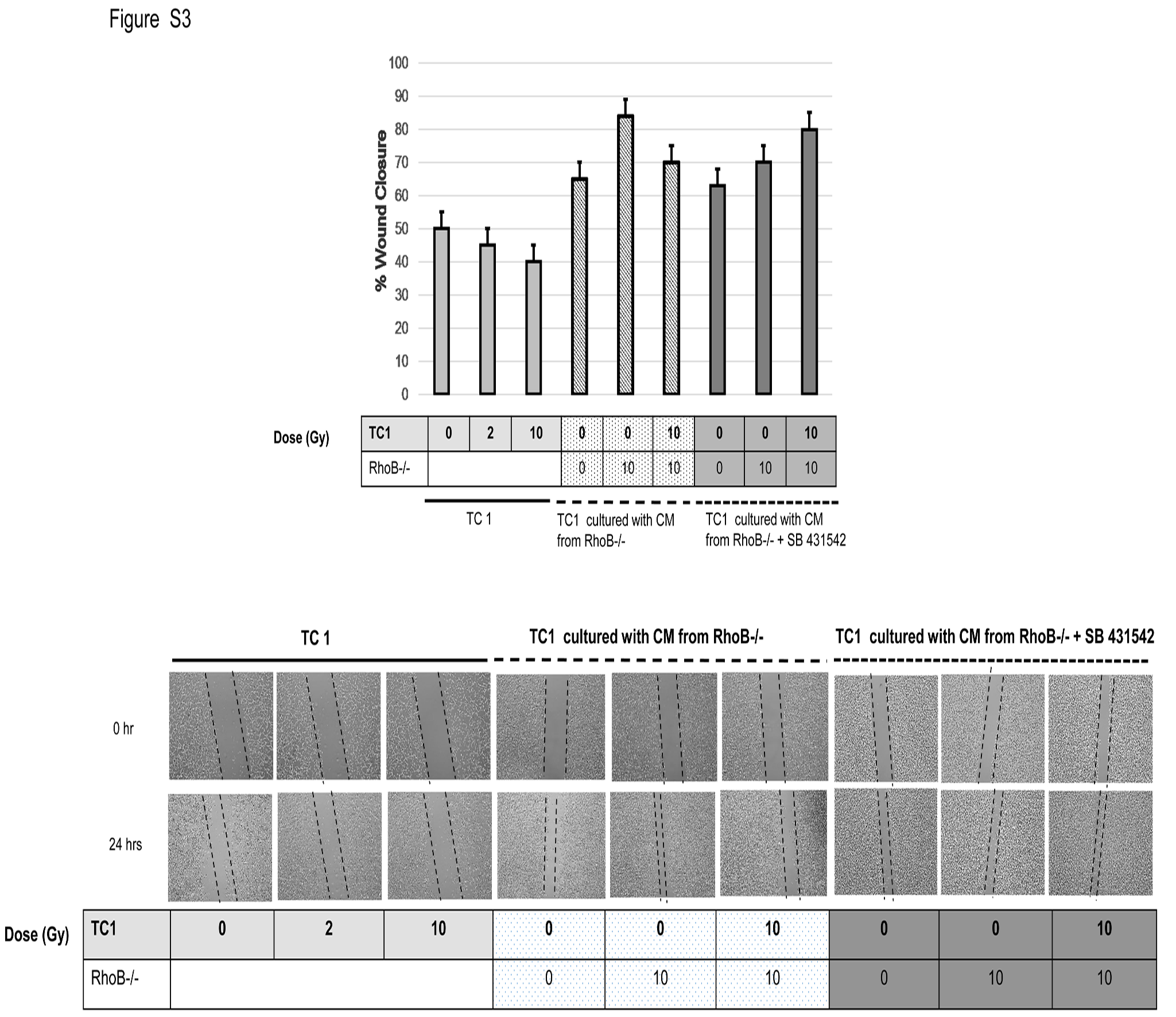

Supplement: S3 Fig — Migratory potential of TC-1 cells was analyzed and quantified in various culture conditions at 0 and 24 Hrs after wounding. Culture conditions are: CM from TC-1 non Irradiated (0Gy) and irradiated at 2, 10 Gy alone or cultured with CM from RhoB -/-fibroblasts with SB41542. Images are acquired at x20 with Nikon Phase contrast, Japan. In all cases, differences were considered significant at: * P<0.05; ** P<0.01; *** P<0.001. (TIF) [file pone.0115447.s003.tif]
